# Microbial Community Dynamics Driven by Different Nitrogen Sources During Forestry Waste Composting for *Pleurotus ostreatus* Cultivation

**DOI:** 10.3390/foods15061084

**Published:** 2026-03-20

**Authors:** Shiqi Li, Yu Liu, Yuan Guo, Dianpeng Zhang, Shoumian Li, Yueyuan Wu, Caige Lu, Qinggang Song, Shouxian Wang, Shuang Song

**Affiliations:** 1 Beijing Engineering Research Center for Edible Mushroom, Institute of Plant Protection, Beijing Academy of Agriculture and Forestry Sciences, Beijing 100097, China; lishiqi0602@163.com (S.L.);; 2 College of Plant Science and Technology, Beijing University of Agriculture, Beijing 102206, China; 3 College of Horticulture, Hebei Agricultural University, Baoding 071000, China

**Keywords:** forestry waste, nitrogen sources, *Pleurotus ostreatus*, lignocellulose degradation, microbial communities

## Abstract

Bioconversion of lignocellulosic biomass into edible, nutrient-rich products using low-cost forestry waste offers substantial ecological and economic benefits. Composting forestry waste as a substrate for oyster mushroom (*Pleurotus ostreatus*) cultivation is an effective recovery strategy. However, the specific microbial-driven mechanisms by which nitrogen sources regulate lignocellulose degradation and compost quality during forestry waste composting for *Pleurotus ostreatus* substrate preparation remain to be elucidated. We evaluated three organic nitrogen sources (bran, soybean meal, and chicken manure) and one inorganic source (diammonium phosphate, DAP) during composting of forest-waste-based substrates. Composting performance and cultivation outcomes were assessed using physicochemical analyses, lignocellulose degradation measurements, high-throughput sequencing of bacterial 16S rRNA and fungal ITS, and biological efficiency. Organic nitrogen sources enhanced compost temperature and lignocellulose degradation by providing sustained nitrogen release, promoting stable colonization of core microbial communities and cooperative bacteria–fungi networks. In contrast, inorganic nitrogen resulted in slower heating, minimal lignocellulose degradation (0.75%), and unstable, competition-dominated microbial networks. Nitrogen sources indirectly shaped microbial communities by regulating the C/N ratio, pH, and electrical conductivity. Lignocellulose degradation and bacterial diversity significantly influenced mushroom biological efficiency, with bacterial diversity strongly regulating degradation rates. The forest waste–bran treatment achieved the highest biological efficiency (78.35%). These findings offer a practical strategy for optimizing forestry waste bioconversion into fungal protein.

## 1. Introduction

The oyster mushroom (*Pleurotus ostreatus*) is one of the major species in large-scale global edible mushroom production, accounting for more than 16% of worldwide mushroom output [1,2]. As the world’s leading producer of *P. ostreatus*, China recorded a total production of 6.25 million tons in 2024. *P. ostreatus* exhibits exceptional environmental adaptability and can acclimate to diverse climatic conditions across regions. It also possesses a strong substrate-degrading capacity, enabling it to utilize a wide range of agricultural and forestry organic materials to sustain growth and metabolism [3]. Moist-heat sterilization and composting are the primary methods used to prepare substrates for *P. ostreatus* cultivation. Compared with moist-heat sterilization, composting offers several advantages, including lower equipment and energy costs, broader applicability of raw materials, and a reduced contamination rate during high-temperature seasons. Consequently, composting has been widely adopted in *P. ostreatus* cultivation worldwide [4,5].

The large-scale development of the *P. ostreatus* industry has generated an urgent demand for cultivation substrates, while simultaneously creating broad prospects for the efficient utilization of agricultural and forestry wastes as substrate materials. *P. ostreatus* is a typical white-rot fungus. During growth, its mycelia secrete lignocellulose-degrading enzymes, such as laccase and manganese peroxidase [6], which break down lignocellulose—otherwise it is difficult to utilize directly—into small-molecule nutrients required for fungal growth, thereby enabling the biological conversion of raw materials [1]. Additionally, *P. ostreatus* possesses dual characteristics as both a saprophytic fungus and a secondary decomposer, forming a synergistic microbial ecosystem with bacteria and other fungi within the compost substrate [7,8]. Through this highly efficient bioconversion process, *P. ostreatus* converts biomass from agricultural and forestry wastes into high-value fungal protein, which not only enhances the resource utilization of waste materials but also provides a nutritional foundation for large-scale cultivation [9,10].

Forestry waste represents a vast and widely distributed biomass resource with a continuously increasing annual output. As a major forestry resource nation, China had approximately 350 million tons of available forestry waste as of 2020. However, the rate of resource utilization remains far from adequate. Large quantities of such waste are discarded or left to decompose naturally, which not only results in the loss of valuable biomass resources but also poses potential environmental pressures [11,12]. Notably, the valorization of forestry waste from legally compliant sources for commercial edible fungus cultivation aligns with regulated forestry management systems and circular development paradigms [13]. This strategy mitigates ecological risks arising from unregulated harvesting of forest residues in unmanaged habitats, including biodiversity loss, disrupted nitrogen cycling, and diminished soil carbon sequestration capacity, while simultaneously achieving dual benefits of economic viability and environmental sustainability [14,15].

Forestry waste is rich in lignocellulose, and its complex chemical structure makes it recalcitrant to direct microbial degradation, thereby constituting a key bottleneck limiting its effective utilization [16,17]. As an environmentally friendly and sustainable biomass conversion technology, composting can overcome the recalcitrance of lignocellulose through synergistic microbial metabolism. Furthermore, it optimizes substrate nutrient composition, enriches functional microorganisms such as fungi and bacteria, and enhances lignocellulose degradation and conversion, thereby providing an effective pathway for the utilization of forestry waste [18,19]. Using forestry waste for composting to produce oyster mushroom cultivation substrates not only reduces production costs but also enables the efficient biological conversion of forestry waste.

Due to inherent limitations, including nitrogen deficiency and a high C/N ratio, forestry waste used directly as a cultivation substrate fails to meet the nutritional balance requirements for *P. ostreatus* mycelial growth. This often results in inefficient composting and impaired mycelial colonization, ultimately compromising yield and quality [16,20]. As a core regulatory factor governing nutrient dynamics during composting, nitrogen source type directly modulates the composition and functional activity of microbial communities. This, in turn, determines compost maturity and the nutrient supply capacity of the substrate [1,21], ultimately affecting compost quality and the subsequent cultivation performance of *P. ostreatus* [22]. Although previous studies have examined the use of forestry waste in edible fungus cultivation, most have focused on adjusting the addition rates of forestry residues or optimizing process parameters. Significant research gaps remain regarding systematic investigations into how different nitrogen sources regulate microbial community structure during composting, influence lignocellulose degradation efficiency, and ultimately promote the bioconversion of forestry waste into *P. ostreatus* cultivation substrates [23,24]. Compared with prior studies, the present study further clarifies the microbial community dynamics and network stability during forestry waste composting driven by different nitrogen sources, revealing the underlying mechanisms by which nitrogen sources influence oyster mushroom cultivation efficiency. Elucidating the regulatory mechanisms of different nitrogen sources can not only alleviate the low bioconversion efficiency caused by nitrogen deficiency in forestry waste but also provide a theoretical basis for the targeted optimization of composting processes, thereby enhancing the resource utilization efficiency of forestry waste [25,26].

Based on the foregoing background, this study aimed to monitor key physicochemical properties, dynamic changes in lignocellulose degradation, and shifts in microbial community structure during the composting of forestry waste amended with different nitrogen sources. By integrating the cultivation performance of *P. ostreatus*, this study further evaluated the effects of nitrogen source type on cultivation substrate quality.

## 2. Materials and Methods

### 2.1. Materials

The forestry waste used in this study was sourced from the Fangshan District, Beijing, China, and consisted of poplar branches (*Populus tomentosa* Carr.) obtained through manual pruning during autumn and winter in a plain afforestation region. After pruning, the branches were air-dried naturally and then ground using a pulverizer into particles measuring 2–3 cm in length for subsequent use. Organic nitrogen sources included bran, soybean meal, and chicken manure, all of which were obtained from Zhuozhou City, Hebei Province, China. The inorganic nitrogen source was diammonium phosphate, purchased from Fangshan District, Beijing, China. The *P. ostreatus* strain was collected and preserved in the Edible Fungi Germplasm Resource Library of the Beijing Academy of Agriculture and Forestry Sciences (Beijing, China) under strain number JZB2101712.

### 2.2. Preparation of the Composting Substrate

Forestry waste sawdust was soaked for 48 h to ensure complete water absorption. Dry chicken manure was pre-wetted after the removal of impurities, whereas diammonium phosphate was completely dissolved in water. Using forestry waste as the base material, different nitrogen sources were added to prepare composting substrates, resulting in four treatments: (1) FWB: 78% forestry waste, 20% wheat bran, and 2% lime; (2) FWSM: 78% forestry waste, 20% soybean meal, and 2% lime; (3) FWCM: 78% forestry waste, 20% chicken manure, and 2% lime; and (4) FWDAP: 92% forestry waste, 5% diammonium phosphate, and 2% lime. Raw materials were weighed according to the prescribed proportions and mixed thoroughly to achieve an initial moisture content of approximately 60%. The composting materials were then piled into circular heaps with a diameter of 1.5 m and a height of 1.0 m. Multiple holes, 30–50 cm deep, were drilled into the pile surface using wooden sticks with a diameter of 5 cm to facilitate aeration. The compost was turned every 2 days. Composting was terminated when the temperature ceased to rise, the color darkened, the texture softened, and no ammonia odor was detected. The total composting period was 6 days. Three replicates were established for each composting treatment.

### 2.3. Cultivation of P. ostreatus

The substrate was packed into polypropylene bags (17 × 33 × 0.05 cm), with 500 g (dry weight) per bag. Activated *P. ostreatus* strain JZB2101712 was inoculated and incubated at 25 °C, with contamination monitored regularly. After complete mycelial colonization of the cultivation bags, they were transferred to a solar greenhouse maintained at 25 °C and 90% relative humidity. Fruiting bodies were harvested, and biological efficiency was calculated as follows: biological efficiency = (fresh weight of fruiting bodies/dry weight of substrate) × 100%.

### 2.4. Physical and Chemical Properties of the Compost Substrate

Composting temperatures at the top, middle, and bottom of each treatment pile were measured at the same time each day, with sampling points selected randomly. Samples were sieved using a reciprocating vibrating sieve with mesh sizes of 4 mesh (4.75 mm), 10 mesh (2.00 mm), and 35 mesh (0.50 mm). The mass of each particle-size fraction was weighed and expressed as a percentage of the total sample mass. Samples were leached with deionized water at a sample-to-water ratio of 1:5 for 8 h. The pH and electrical conductivity (EC) of the supernatant were then measured using a pH meter (PB-10, Sartorius) and a conductivity meter (DDS-320, Shanghai INESA Scientific Instruments Co., Ltd., Shanghai, China), respectively. Dried sample (1 g) was placed in a 10 mL centrifuge tube, to which 5 mL of deionized water was added. After leaching at room temperature for 24 h, the mixture was filtered. The filter residue was drained for 12 h, and its wet weight was recorded. Water-holding capacity (WHC) was calculated using the formula: WHC = (wet weight of residue − dry weight of sample)/wet weight of residue. A centrifuge tube of known volume (V) was filled with dried substrate in its natural packing state, and bulk density (BD, g/cm^3^) was calculated as BD = G/V, where G represents the mass of the sample. For each treatment, the total nitrogen (TN) and organic matter (OM) contents of substrate samples were determined at three time points: the first day of composting (D1), the end of composting (D6), and the period of complete mycelial colonization (M). TN was determined using the Kjeldahl method, while OM content was analyzed by the potassium dichromate volumetric method. OM content was derived from the measured organic carbon content using the conversion equation OM = organic carbon × 1.724, and the carbon-to-nitrogen (C/N) ratio was subsequently calculated as the ratio of organic carbon to total nitrogen. The contents of lignin, cellulose, and hemicellulose in substrate samples collected at D1, D6, and M were determined according to a previously reported method [27].

### 2.5. Changes in Microbial Communities

Total genomic DNA was isolated from compost substrate samples with the E.Z.N.A.^®^ Soil DNA Kit (Omega Bio-tek, Norcross, GA, USA). The integrity of the isolated genomic DNA was verified by 1% agarose gel electrophoresis, while the DNA concentration and purity were quantified with a NanoDrop 2000 spectrophotometer (Thermo Fisher Scientific, Wilmington, DE, USA). To analyze the bacterial community composition, the V3–V4 hypervariable region of the 16S rRNA gene was PCR-amplified with primers 338F (5′-ACTCCTACGGGAGGCAGCAG-3′) and 806R (5′-GGACTACHVGGGTWTCTAAT-3′), each of which carried unique barcode sequences [28]. For fungal community profiling, the internal transcribed spacer (ITS) region was amplified with barcode-labeled primers ITS1F (5′-CTTGGTCATTTAGAGGAAGTAA-3′) and ITS4R (5′-TCCTCCGCTTATTGATATGC-3′) [29]. Each PCR amplification system was prepared with a total volume of 20-μL, containing 4 μL of 5× FastPfu buffer, 2 μL of 2.5 mM dNTPs, 0.8 μL of each primer (5 μM), 0.4 μL of FastPfu DNA polymerase, 10 ng of template DNA, and DNase-free water to reach the final volume. The PCR cycling parameters were set as follows: an initial denaturation at 95 °C for 3 min; followed by 27 cycles comprising denaturation at 95 °C for 30 s, annealing at 60 °C for 30 s, and extension at 72 °C for 45 s; and a final extension at 72 °C for 10 min, followed by a hold at 4 °C. All amplifications were carried out with a T100 Thermal Cycler (Bio-Rad, Hercules, CA, USA). Following electrophoretic validation of the PCR products, they were purified using AMPure^®^ PB beads (Pacific Biosciences, Menlo Park, CA, USA) and quantified using a Qubit 4.0 fluorometer (Thermo Fisher Scientific, Waltham, MA, USA). High-throughput sequencing was conducted on the PacBio Sequel IIe System. Based on a 97% sequence similarity cutoff, effective sequences were clustered into operational taxonomic units (OTUs) using UPARSE software (v7.1). Bacterial 16S rRNA gene sequences were annotated against the SILVA database (v138), while fungal ITS sequences were compared against the UNITE database (v9.0) to achieve taxonomic annotation at the phylum, class, order, family, genus, and species levels. All sequencing procedures were completed by Majorbio Bio-Pharm Technology Co., Ltd. (Shanghai, China).

### 2.6. Statistical Analysis

Experimental data were processed using Microsoft Excel 2016, and one-way analysis of variance (ANOVA) was performed using SPSS version 22.0. Alpha diversity indices were calculated with the mothur software (v1.48.1) [30], and intergroup differences were assessed using the Wilcoxon test. Principal coordinate analysis (PCoA), based on the Bray–Curtis distance, was used to evaluate similarities in microbial community structure among samples. The non-parametric PERMANOVA was applied to evaluate whether differences in microbial community structure among samples were statistically significant. Linear discriminant analysis effect size (LEfSe) analysis [31] was used to identify taxa with significantly different abundances among groups, from the phylum to genus levels (logarithmic LDA score > 2, *p* < 0.05). Based on the OTU detection rate in each sample, OTUs were classified into three categories: persistent (detection rate > 80%), intermittent (20% < detection rate < 80%), and transient (detection rate < 20%) [32]. Microbiome co-occurrence network analysis was performed using the R package WGCNA, based on Pearson correlation coefficients with thresholds of *ρ* > 0.8 and *p* < 0.01. Heatmaps of Mantel test results were generated using the R package vegan. Structural equation modeling (SEM) was conducted using IBM SPSS Amos version 24.

## 3. Results and Discussion

### 3.1. Changes in Physical and Chemical Properties of Substrate During Composting

**Figure 1:**
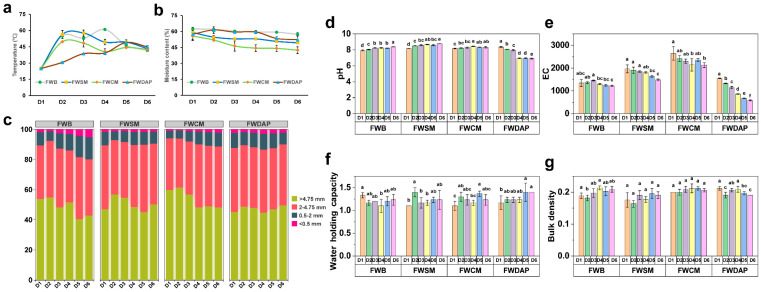
Variations in physicochemical properties of the substrate during the composting process. FWB, forestry waste + bran; FWSM, forestry waste + soybean meal; FWCM, forestry waste + chicken manure; FWDAP, forestry waste + diammonium phosphate. (**a**) Temperature; (**b**) moisture content; (**c**) particle size distribution; (**d**) pH; (**e**) electrical conductivity (EC); (**f**) water-holding capacity; (**g**) bulk density. Different lowercase letters indicate significant differences (*p* < 0.05).

The physicochemical properties of the composting process are key indicators of composting performance and final product quality [33]. Temperature serves as a critical parameter during composting and plays a vital role in shaping the succession of microbial communities [34]. The compost temperatures of the four treatments (FWB, FWSM, FWCM, and FWDAP) exhibited a pattern of initial increase followed by a gradual decrease, with maximum temperatures occurring during the middle stage of composting (Figure 1a). During the early stage, microbial metabolic activity was rapidly activated, and microorganisms—particularly thermophilic taxa—decomposed readily degradable organic matter. These thermophilic microorganisms exhibited intense metabolic activity and high rates of heat production, driving the rapid temperature increase in each pile [35]. Among the four treatments, FWB and FWSM showed the fastest temperature increase, followed by FWCM, whereas FWDAP exhibited the slowest rise. Notably, the maximum temperature of FWDAP did not exceed 50 °C. As organic matter was progressively consumed, microbial metabolic activity gradually declined, resulting in a corresponding decrease in temperature across all treatments. By the end of composting (D6), the temperatures of all piles had stabilized, indicating the completion of the primary composting stage. These results demonstrate that organic nitrogen sources effectively promote rapid temperature increases in composting piles, consistent with the findings of Li et al. [36]. When comparing the composting performance of rice straw amended with an inorganic nitrogen source (urea) and an organic nitrogen source (protein hydrolysate of leather, PHL), the PHL treatment exhibited a faster initial temperature increase. The addition of the organic nitrogen source significantly enhanced aerobic microbial activity, thereby accelerating organic matter degradation and promoting the release of substantial amounts of heat energy.

During the composting process, the moisture content of all treatments exhibited a decreasing trend (Figure 1b). Moisture content in the FWB and FWSM treatments declined gradually, whereas the FWDAP treatment showed slight fluctuations. The FWCM treatment experienced the highest rate of moisture loss, which may be attributed to the physical structure of chicken manure. The high content of undigested crude fiber in chicken manure created and maintained numerous aeration pores. Compared with wheat bran, soybean meal, and diammonium phosphate, these pores provided more efficient pathways for water evaporation. This observation is consistent with the findings of Liu et al. [37], who reported that during the composting maturity stage, moisture reduction rates of substrates with different particle sizes varied significantly, with higher porosity accelerating water evaporation. Moisture content is a critical factor influencing the quality of mushroom substrates and the biological efficiency of fruiting bodies at later stages. The optimal moisture content of a compost pile should be maintained at approximately 60%. Excessively high moisture content can lead to water accumulation due to the limited water-holding capacity of the primary material, sawdust, which in turn induces anaerobic microbial respiration and delays the increase in pile temperature. Conversely, excessively low moisture levels inhibit microbial activity and ultimately compromise substrate composting [38].

The particle size composition of the composting substrates in each treatment exhibited a consistent pattern (Figure 1c). Particles larger than 4.75 mm constituted the largest proportion of the substrate and served as the dominant particle component of the compost piles, followed by particles sized 2–4.75 mm. In contrast, the proportion of particles smaller than 2 mm was relatively low and showed minimal variation throughout the composting process. The predominance of large particles not only helps maintain pore structure and aeration within the piles but also provides a favorable environment for microbial metabolism [39]. Unlike the evaluation criteria applied to smaller particle sizes in mature compost [40], larger particle sizes in substrates are more conducive to mushroom mycelium colonization and to the absorption and utilization of oxygen, nutrients, and moisture.

The pH of all treatments was maintained within a range of 7.0–9.0 throughout the composting process (Figure 1d). This stability can be attributed to the incorporation of 2% lime in the substrate formulation. The alkaline buffering capacity of lime effectively counteracted pH changes induced by microbial metabolites, thereby maintaining a relatively stable chemical environment. The pH of the three treatments supplemented with organic nitrogen sources showed only minor fluctuations, with a slight increase during the later stages of composting. In contrast, the pH of the FWDAP treatment decreased significantly as composting progressed. When microorganisms utilize organic nitrogen sources, the decomposition of readily degradable organic nitrogen enhances ammonia release. In addition, certain microbial populations may produce alkaline antibiotics, which can lead to an increase in pH [41,42]. By contrast, when inorganic nitrogen sources are utilized, microorganisms generate H^+^ through nitrification (i.e., the oxidation of NH_4_^+^ to NO_3_^−^), resulting in a decrease in pH [43].

During the composting process, the FWCM treatment exhibited the highest EC among all treatments, with values ranging from 2133.22 to 2643.22 μS/cm. In contrast, the EC values of the FWB, FWSM, and FWDAP treatments remained at moderate levels, ranging from 500 to 2000 μS/cm. Overall, EC values across all treatments showed a gradual decreasing trend as composting progressed, although slight fluctuations were observed in treatments such as FWB and FWCM (Figure 1e). This pattern is consistent with the findings of Li et al. [44]. During the early stage of composting, microorganisms actively decompose organic matter, releasing substantial amounts of organic acids and soluble ions, which can result in a temporary increase in EC. As microbial communities succeed over time, organic acids are progressively degraded, and some soluble ions are lost through water leaching, leading to a subsequent decrease in EC values [45].

The water-holding capacity of the four treatments fluctuated between 1.1 and 1.4 throughout the composting process, without exhibiting a clear or consistent pattern of change (Figure 1f). Among the treatments, FWDAP maintained a relatively high water-holding capacity across all composting stages. This may be attributed to the use of inorganic nitrogen sources; in contrast to organic nitrogen sources, these do not promote excessive decomposition of the fiber structure driven by intense microbial metabolism. In addition, the FWDAP treatment exhibited a slower temperature increase within the compost pile, which reduced high-temperature drying and prevented structural collapse of the substrate. This preserved sufficient pore space for water adsorption, thereby enhancing overall water-holding capacity.

The bulk density of all treatments remained between 0.1 and 0.2 throughout the composting process (Figure 1g), indicating that the substrate pore structure was maintained within an optimal range. This condition ensured adequate oxygen availability for aerobic microbial metabolism [46]. Moreover, these bulk density values meet the structural requirements for subsequent *P. ostreatus* cultivation substrates, facilitating mycelial colonization and growth.

### 3.2. Changes in C/N Ratio and Lignocellulose Content of the Substrate During Composting Process

The C/N ratio is a critical factor regulating the composting process [47,48]. Efficient microbial metabolism depends on a balanced supply of carbon and nitrogen [27]. Significant differences in organic matter and nitrogen contents were observed among the four nitrogen sources used in this study (Table S1), resulting in variations in the initial C/N ratios of the four treatments (Table S2). Because the C/N ratio of forestry waste—the primary raw material—was as high as 101.99, the initial C/N ratios of all treatments exceeded the optimal range (25–30:1) for composting [49]. An excessively high C/N ratio reduces the heat-generating capacity of microorganisms, leading to inadequate substrate maturation [50]. In the FWB and FWSM treatments, which had relatively high nitrogen availability, microorganisms preferentially utilized these readily accessible nitrogen sources, resulting in an increasing trend in the C/N ratio during the later stages of composting. In contrast, microorganisms in the FWCM and FWDAP treatments consumed a greater proportion of carbonaceous components, leading to a decrease in the C/N ratio by the end of composting. During the fully colonized mycelial stage, lignocellulosic components of the substrate were extensively decomposed and utilized by *P. ostreatus*, resulting in lower C/N ratios across all treatments compared with those observed at the end of composting. The C/N ratios of the FWB, FWSM, and FWCM treatments decreased by 30.55%, 31.42%, and 30.93%, respectively, during the mycelial growth phase. In contrast, the FWDAP treatment exhibited lower carbon and nitrogen utilization efficiency at this stage, as indicated by a smaller reduction in the C/N ratio (10.93%) relative to the end of composting. Notably, the final C/N ratio of the composting substrate in this study remained higher than the previously reported optimal range (28:1–30:1) for *P. ostreatus* cultivation [21]. An excessively high C/N ratio (>35:1) markedly suppresses the activity of lignocellulolytic enzymes, such as cellulase and laccase, resulting in a reduction in more than 40% in lignocellulose degradation efficiency [51,52]. In addition, substrates with high C/N ratios fail to supply sufficient nitrogen for mycelial growth, thereby slowing mycelial growth rates and producing sparse, thin mycelia. These observations are consistent with findings reported in subsequent studies.

**Figure 2:**
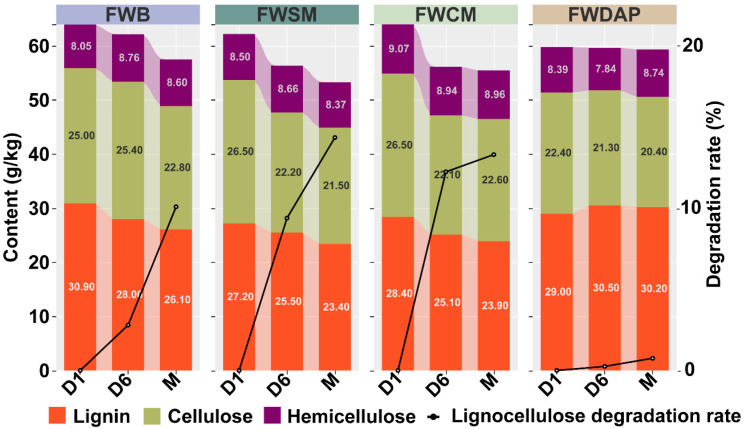
Lignocellulose content and lignocellulose degradation rates of the substrate on composting day 1 (D1), composting day 6 (D6), and at the mycelium fully colonized stage (M).

During the composting process, the contents of lignin, cellulose, and hemicellulose in all treatments decreased continuously (Figure 2), indicating ongoing lignocellulose degradation during both the composting and mycelial growth stages. The lignocellulose degradation rates in the FWB, FWSM, and FWCM treatments increased progressively over time. During the composting phase, the FWCM treatment exhibited the highest lignocellulose degradation rate (12.24%). In contrast, during the mycelial growth phase, *P. ostreatus* mycelia in the FWB treatment utilized the greatest proportion of lignocellulose (7.29%). However, the lignocellulose degradation rate in the FWDAP treatment remained low throughout the process, with only 0.75% of the lignocellulose consumed by the end of mycelial colonization. This limited degradation may be attributed to the composting temperature of the FWDAP treatment, which did not exceed 50 °C. In aerobic composting systems, the microbial metabolic activities responsible for cellulose and hemicellulose degradation generally require higher temperatures (often exceeding 60 °C), whereas temperatures around 50 °C are more favorable for lignin degradation [22]. Lignin degradation is considered the rate-limiting and critical step in the overall biodegradation of lignocellulose [53]. Previous studies have shown that during the composting of mushroom cultivation substrates, the activities of lignin-degrading enzymes, such as laccase and manganese peroxidase, increased with prolonged composting time and reached their maximum levels at the end of the composting process [52,54]. In the present study, lignin degradation in the FWB, FWSM, and FWCM treatments occurred primarily during the composting stage, whereas the lignin degradation rate decreased slightly during the mycelial growth phase. This pattern is attributed to extensive lignin degradation by bacteria, including *Pseudomonas* and *Streptomyces*, together with various white-rot fungi during composting [55,56]. When *P. ostreatus* mycelia were inoculated into the substrate and rapidly colonized it to become the dominant fungus, primary metabolism predominated during the vegetative growth phase, resulting in reduced secretion of lignin-degrading enzymes [57]. The results indicate that different nitrogen sources exert significant effects on the C/N ratio and on lignocellulose degradation. Compared with inorganic nitrogen sources, organic nitrogen sources were more conducive to lignocellulose degradation.

### 3.3. Bacterial Community Composition During Composting

**Figure 3:**
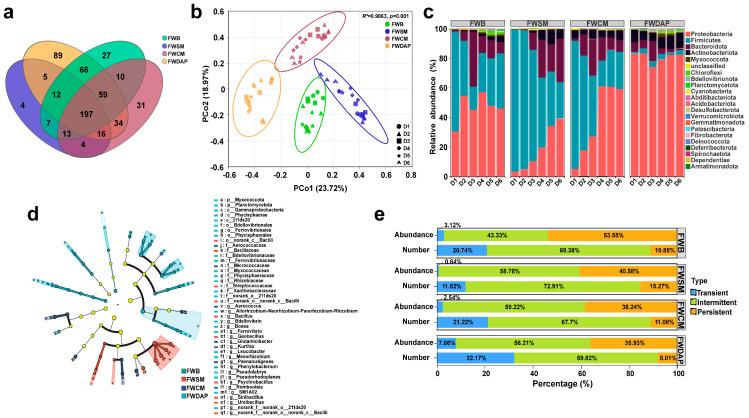
Comparison of bacterial community structures among different treatments. (**a**) Venn diagram of bacterial operational taxonomic units (OTUs) among treatments; (**b**) principal coordinates analysis (PCoA) of bacterial communities at the OTU level based on Bray–Curtis distance. Ellipse boundaries represent 95% confidence intervals for the corresponding treatments; (**c**) relative abundance at the phylum level; (**d**) LEfSe analysis of bacterial communities among treatments (*p* < 0.05, LDA score ≥ 2.0). Green, red, dark blue and light blue nodes represent microbial taxa that are significantly enriched in the corresponding group; (**e**) environmental sensitivity classification of bacterial communities.

The number of operational taxonomic units (OTUs) and intergroup overlap patterns among the four treatments were evaluated using Venn diagrams [58]. A total of 197 bacterial OTUs were shared across all 4 treatments. The FWDAP treatment exhibited the highest number of unique OTUs (89), whereas the FWSM treatment contained only 4 unique OTUs (Figure 3a). Microbial community richness and diversity were further assessed using alpha diversity indices [59]. At the initial stage of composting, the Shannon and Chao1 indices were highest in the FWDAP treatment (Table S3), showing significant differences compared with the three organic nitrogen source treatments (*p* < 0.05). However, as composting progressed, bacterial richness and diversity gradually increased in each organic nitrogen source treatment. By the end of composting, the richness of the FWDAP treatment showed no significant difference compared with the FWB and FWCM treatments.

Principal coordinates analysis (PCoA) based on Bray–Curtis distance provided a visual representation of differences in bacterial community composition among treatments. The addition of different nitrogen sources significantly influenced bacterial community structure during composting (Figure 3b). The first two principal coordinates (PCo1 and PCo2) explained 23.72% and 18.97% of the total variation in bacterial communities, respectively, collectively accounting for 42.69% of the observed variation in community structure. The four treatments formed distinct clusters and were significantly separated (PERMANOVA, *p* = 0.001), indicating that the addition of different nitrogen sources was the primary driver of variation in microbial community structure. In addition, the D1 and D6 sampling points within each treatment were significantly separated, indicating substantial changes in bacterial community structure over the course of composting.

Phylum-level analysis of bacterial communities revealed that Proteobacteria, Firmicutes, Bacteroidota, and Actinobacteriota were the dominant phyla across all four treatments (Figure 3c). The high abundance of these phyla was consistent with their functional roles in carbon and nitrogen metabolism. Actinobacteriota represent a key functional group involved in lignocellulose degradation, and their abundance distribution can preliminarily indicate the substrate degradation potential of different treatments. This phylum plays a critical role in organic matter decomposition by promoting the synthesis of lignocellulolytic enzymes, thereby enhancing the efficiency of organic matter degradation [60,61]. Proteobacteria represented the core functional group participating in nitrogen transformation, driving key processes such as nitrification and denitrification to regulate the nitrogen cycle in the compost system [62]. Firmicutes play an important role in cellulose decomposition and utilization during composting [63]. This phylum can increase compost temperature by enriching thermotolerant strains, thereby inhibiting the proliferation of pathogenic bacteria and improving the overall substrate quality, which creates a favorable microenvironment for the subsequent mycelial growth of *P. ostreatus.* Bacteroidota exhibit a strong ability to degrade organic matter and provide essential energy for the proliferation and metabolism of microbial communities during the initial stage of composting [64].

During composting, the relative abundance of Proteobacteria in the organic nitrogen source treatment groups (FWB, FWSM, and FWCM) gradually increased as the process progressed. In contrast, the relative abundances of Firmicutes and Bacteroidetes exhibited a dynamic declining trend, whereas the abundance of Actinobacteria continued to increase. Previous studies have also shown that Proteobacteria and Firmicutes are dominant phyla in composting systems based on sugarcane straw substrates for oyster mushroom cultivation [65]. These two phyla play core roles in lignocellulose degradation and nitrogen transformation during the thermophilic phase of composting [27]. In contrast, in the FWDAP treatment group (inorganic nitrogen source), Proteobacteria consistently remained the dominant phylum with a stable relative abundance, whereas Firmicutes maintained a comparatively low abundance. This divergence in community structure indicates that nitrogen source type exerts a directional selective effect on dominant bacterial phyla through nutritional selection [66]. From the perspective of composting phases, rapidly proliferating microbial groups specializing in the utilization of easily degradable organic matter—such as Firmicutes—accounted for a relatively high proportion during the early stage (D1). By the late composting stage (D6), groups with metabolic functions targeting refractory substrates, such as Proteobacteria, gradually became dominant, with their relative abundances increasing progressively. The high abundance of core degradative functional groups, such as Actinobacteriota, in the FWSM treatment was consistent with its higher lignocellulose degradation rate during the early stage. In the FWDAP treatment, although Proteobacteria were the most abundant phylum, this group was more strongly associated with nitrogen transformation and the metabolism of readily degradable organic matter [67]. In contrast, the abundance of core lignocellulose-degrading groups, such as Actinobacteria, was low, resulting in reduced functional redundancy related to lignocellulose degradation and a weaker substrate degradation capacity.

LEfSe analysis was applied to identify microbial biomarkers contributing to intergroup differences [68]. Different nitrogen source treatments significantly altered bacterial community composition during composting, resulting in the identification of 24 statistically distinct biomarkers (LDA score ≥ 2, *p* < 0.05). At the phylum level, Myxococcota and Planctomycetota emerged as significantly differentially enriched core phyla across treatments, with both showing enrichment in the FWDAP treatment. At the genus level, *Phenylobacterium* was the only key genus enriched in the FWB treatment. The FWSM and FWCM treatments harbored six and five differentially enriched genera, respectively, whereas the FWDAP treatment exhibited significantly higher proportions of functional taxa belonging to 11 genera. These differentially abundant taxa reflect the selective effect of nitrogen source type on bacterial community composition. *Planctomycetota*, which was enriched in the FWDAP treatment, is well known for its metabolic capacity to utilize NH_4_^+^ (ammonium) as an electron donor and NO_2_^−^ (nitrite) as an electron acceptor to produce N_2_ under anaerobic conditions [69,70]. Such bacteria can rapidly exploit readily available nitrogen sources, thereby driving key nitrogen cycling processes, including nitrification. In contrast, treatments supplied with organic nitrogen sources enriched *Bacillus*, *Geobacillus*, and other bacteria possessing both organic matter–degrading and thermotolerant capabilities, making them well suited for the degradation of complex organic nitrogen compounds [71,72,73].

Based on environmental sensitivity and detection frequency, microorganisms can be classified into transient, intermediate, and persistent taxa. Figure 3e illustrates the relative abundance and proportional representation of each bacterial group across different treatments. Intermediate taxa accounted for 68.38%, 72.91%, 67.70%, and 59.82% of the communities in the four treatments, respectively, indicating that bacterial community diversity during composting was predominantly shaped by stage-adapted groups. The proportion of persistent microorganisms in the organic nitrogen source treatments (FWB, FWSM, and FWCM) was significantly higher than that observed in the FWDAP treatment. These results indicate that the gradual nitrogen release associated with organic nitrogen sources creates a more stable substrate environment, thereby favoring the long-term colonization of functional bacteria and the formation of structurally stable core microbial communities. In contrast, readily available nitrogen supplied by inorganic nitrogen sources promotes rapid microbial proliferation during the early stage of composting. However, environmental conditions fluctuate sharply following nitrogen depletion, making sustained colonization difficult for most bacterial taxa. Consequently, only a limited number of stress-tolerant functional bacteria maintain dominance, leading to low functional redundancy [37,74,75,76].

### 3.4. Fungal Community Composition During Composting

**Figure 4:**
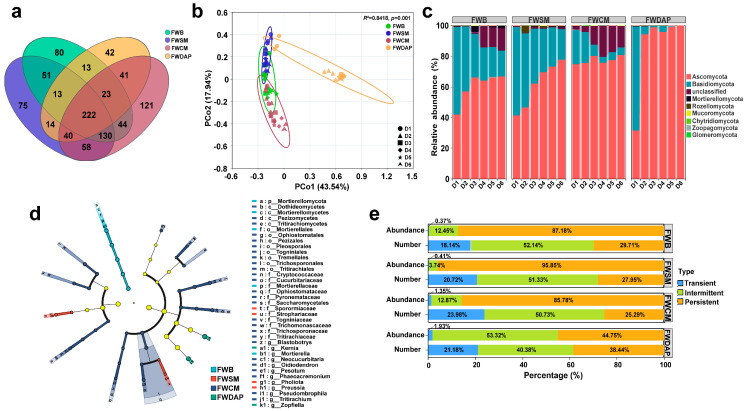
Comparison of fungal community structures among different treatments. (**a**) Venn diagram of fungal OTUs among treatments; (**b**) PCoA of fungal communities at the OTU level based on Bray–Curtis distance. Ellipse boundaries represent 95% confidence intervals for the corresponding treatments; (**c**) relative abundance at the phylum level; (**d**) LEfSe analysis of fungal communities among treatments (*p* < 0.05, LDA score ≥ 2.0). Light blue, red, dark blue and green nodes represent microbial taxa that are significantly enriched in the corresponding group; (**e**) environmental sensitivity classification of fungal communities.

A total of 222 fungal OTUs were shared across the 4 treatments (Figure 4a). The numbers of unique OTUs in the FWB, FWSM, FWCM, and FWDAP treatments were 80, 75, 121, and 42, respectively. Alpha diversity analysis revealed that fungal community richness and diversity in the FWCM treatment were higher than those in the other three treatments during the initial composting phase (Table S4). However, as composting progressed, fungal richness and diversity in the three organic nitrogen source treatments gradually converged. In contrast, the FWDAP treatment significantly suppressed fungal species richness and diversity.

PCoA revealed that the first two principal coordinates (PCo1 and PCo2) explained 43.54% and 17.94% of the variation in fungal community structure, respectively (Figure 4b). After day 2 (D2), samples from the FWDAP treatment formed a distinct cluster that was clearly separated from those of the other three treatments. In contrast, samples from the organic nitrogen source treatments were more tightly clustered, indicating a high degree of similarity in fungal community structure among these treatments. This pattern is consistent with the findings of Zhu et al. [77]. Compared with those in fungi, differences in bacterial community structure were more pronounced, suggesting that bacteria are more sensitive to changes in physicochemical properties during composting [78].

Ascomycota and Basidiomycota were the dominant phyla across all treatments (Figure 4c), with Ascomycota occupying an absolutely dominant position. These two phyla show distinct metabolic and adaptive traits that drive their stage-specific succession during composting. Throughout the composting process, the relative abundance of Ascomycota showed a gradual increasing trend in all four treatments, whereas that of Basidiomycota gradually decreased. Although these two dominant phyla are phylogenetically closely related and exhibit similar community succession dynamics, Basidiomycota dominated the early composting stage, while Ascomycota proliferated extensively during the later stage, progressively increasing in relative abundance and ultimately replacing Basidiomycota as the dominant phylum. Hu et al. [79] also reported that during the co-composting of spent mushroom substrate and swine manure, the relative abundance of Ascomycota reached a high level of 90.3–99.6% during the late composting stage, making it the dominant phylum in the system. Although most Basidiomycota species possess strong lignin-degrading capabilities, their poor tolerance to high temperatures limits their abundance during the thermophilic phase of composting [80]. In contrast, Ascomycota are widely distributed in composting environments; they can not only utilize a wide range of lignocellulosic substrates for metabolic activities but also exhibit strong adaptability to environmental stresses, such as temperature fluctuations and nutrient limitation [81].

The LEfSe analysis of the fungal community (Figure 4d) indicated that Mortierellomycota was the most dominantly differentially enriched phylum in the FWB treatment. The key differentially enriched genera in the FWSM treatment were *Pholiota* and *Preussia*, whereas *Kernia* and *Zopfiella* were the dominantly enriched fungi in the FWDAP treatment. The FWCM treatment harbored the greatest number of core biomarkers, and these fungal taxa are capable of efficiently degrading complex organic compounds, such as humus precursors and protein residues, in chicken manure [82,83,84].

The core taxa of the fungal community are shown in Figure 4e. In the organic nitrogen source treatments, persistent groups accounted for more than 85% of the community, indicating that the stable environment created by the slow mineralization of organic nitrogen sources supported the long-term colonization of core fungal taxa and the establishment of a stable functional community. In contrast, in the FWDAP treatment, the relative abundances of intermediate (53.32%) and persistent groups (44.75%) were comparable, reflecting greater fluctuations in fungal community structure. The dominance of core persistent groups was not pronounced, which corresponded to a lower composting efficiency phenotype. The initial microbial community during composting may exert a lasting influence on the later composting stages, thereby affecting compost maturity and stabilization [85]. Fungi are the primary decomposers of complex polymers during composting, and previous studies have reported that fungal communities may be more sensitive to fluctuations in composting environmental parameters than bacterial communities [81]. However, in the present study, fungal community structures across treatments were more stable than bacterial communities, which may be attributed to lagged responses resulting from differences in physiological metabolism.

### 3.5. Co-Occurrence Network and Mantel Test

**Figure 5:**
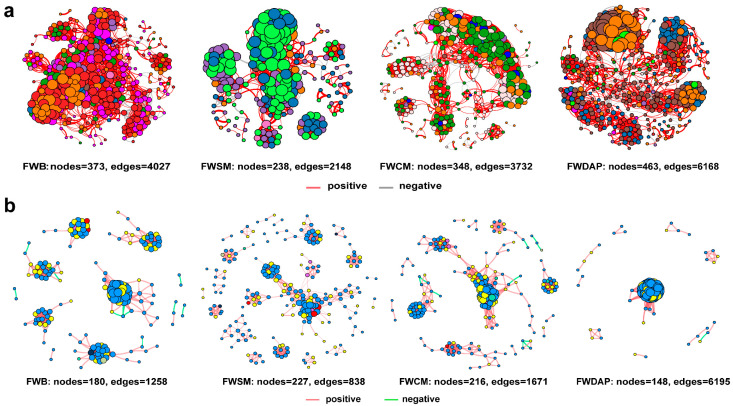
Co-occurrence network of microbial communities at the genus level for different treatments during the composting process, based on Pearson correlation (*ρ* > 0.8, *p* < 0.01). Only modules containing more than two nodes are shown. Node size indicates the number of connections. Different phyla are indicated by different colors. (**a**) bacteria; (**b**) fungi.

Co-occurrence network analysis effectively reveals complex relationships among microbial taxa and has been widely applied to investigate microbial interactions across diverse habitats [86]. To further elucidate microbial co-occurrence patterns in compost substrates, bacterial and fungal co-occurrence networks were constructed for the four treatments. The characteristics of the bacterial co-occurrence networks are presented in Figure 5a and Table S5. The FWB treatment contained 373 nodes and 4027 edges, with densely connected nodes and a high proportion of positive correlations, indicating complex interactions among bacterial taxa. The FWSM treatment contained 238 nodes and 2148 edges, with nodes clustered into multiple distinct modules wherein positive correlations remained predominant. The FWCM treatment harbored 348 nodes and 3732 edges, exhibiting a clearer modular structure wherein nodes of different colors formed independent clusters connected by dense positive correlations. The FWDAP treatment exhibited the highest numbers of nodes (463) and edges (6168), along with the most complex associations and large clusters. These features are closely related to the nutrient supply characteristics of inorganic nitrogen sources: the readily available nitrogen (NH_4_^+^) provided by DAP triggered rapid microbial proliferation during the early stage, leading to a sharp increase in species richness. However, pulsed nitrogen supply induces intense interspecific competition, thereby generating numerous interactions. The fungal co-occurrence networks are shown in Figure 5b and Table S6. The number of nodes in the organic nitrogen source treatments was higher than that in the inorganic nitrogen source treatment, indicating that organic nitrogen sources are more conducive to supporting the involvement of highly diverse fungal communities in network interactions. Although the FWDAP treatment had the fewest nodes, its number of edges (6195) was substantially higher than that of the other treatments (FWCM: 1671; FWB: 1258; FWSM: 838). This pattern indicates more intensive interactions among fungal taxa within this treatment, resulting in tightly interconnected networks. Disturbance to core taxa within this group is likely to trigger functional fluctuations across the entire community [87], which is consistent with the previously observed low composting efficiency and weak community stability in the FWDAP treatment. Several studies have suggested that more tightly connected networks may enhance nutrient utilization efficiency [88]. In this study, the network responses of bacterial and fungal communities to different nitrogen sources exhibited significant complementarity, reflecting the differential regulation of microbial interactions by nitrogen sources. Organic nitrogen sources maintain environmental stability through slow nitrogen mineralization, with fungi and bacteria primarily responsible for complex organic matter degradation and nitrogen transformation or simple organic matter degradation, respectively. This functional synergy contributes to the formation of a stable interactive network [89]. In the inorganic nitrogen source treatment, both bacteria and fungi developed network characteristics characterized by high interaction intensity and low stability, achieved through enhanced interspecific competition to adapt to environmental stress.

**Figure 6:**
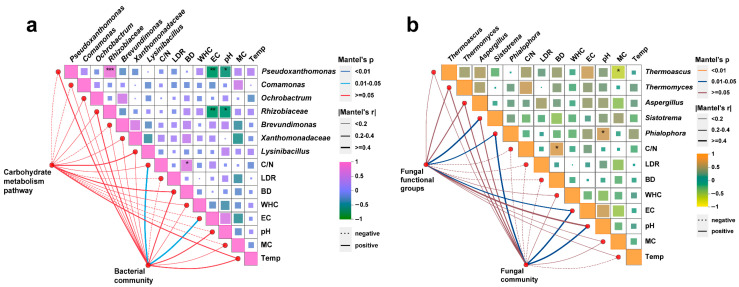
Correlation analysis among bioinformatics parameters, physicochemical environmental factors, and core microorganisms using the Mantel test. The color intensity of the squares indicates the magnitude of the Pearson correlation coefficient. The color intensity and thickness of the lines indicate Mantel test correlations between the microbial matrix and environmental factors. Significant correlations are denoted by “*”, “**” and “***”, which represent *p* < 0.05, *p* < 0.01, and *p* < 0.001, respectively. C/N, C/N ratio; LDR, lignocellulose degradation rate; BD, bulk density; WHC, water-holding capacity; MC, moisture content; Temp, temperature. (**a**) bacteria; (**b**) fungi.

Variations in the composition of predicted bacterial functions during the composting process were analyzed using PICRUSt2 (Figure S1). Among these functions, the metabolic category exhibited the highest relative abundance, accounting for 23.1%—consistent with findings from previous studies [90,91]. For the Mantel test (Figure 6a), carbohydrate metabolism pathways related to substrate degradation, six core bacterial genera with an occurrence frequency greater than 95%, six physicochemical properties, the C/N ratio, and the lignocellulose degradation rate were selected as explanatory variables. The C/N ratio and EC were significantly correlated with bacterial diversity (*p* < 0.05), in agreement with prior research [37,92]. *Pseudoxanthomonas* and *Rhizobiaceae* were significantly negatively correlated with pH and EC, respectively (*p* < 0.05). The fungal Mantel test (Figure 6b) showed that fungal community diversity was significantly correlated with *Phialophora*, EC, and pH (*p* < 0.05). Fungal functional groups were significantly correlated with *Sistotrema*, *Phialophora*, and EC (*p* < 0.05). In addition, *Thermoascus* and *Phialophora* were significantly influenced by moisture content and pH, respectively (*p* < 0.05). Notably, the thermophilic fungus *Thermoascus* is a core functional taxon for organic matter degradation during composting, playing important roles in lignin degradation and the production of thermostable enzymes [93]. The observed correlations between *Thermoascus* and environmental factors clarify the key physicochemical indicators regulating this functional group. Previous studies have reported that dynamic changes in microbial community composition during composting are driven by multiple environmental factors and material transformations [94]. The findings of this study demonstrate that nitrogen sources can regulate microbial community structure during composting by modulating key environmental factors (C/N ratio, pH, and EC). Collectively, nitrogen source types shape the directional selection of microbial functional taxa by mediating the stability of key environmental factors, further constructing microbial co-occurrence networks with divergent structural characteristics, and ultimately regulating the lignocellulose degradation efficiency and quality of the compost substrates.

### 3.6. Effects of Different Nitrogen Sources on Cultivation of P. ostreatus

**Figure 7:**
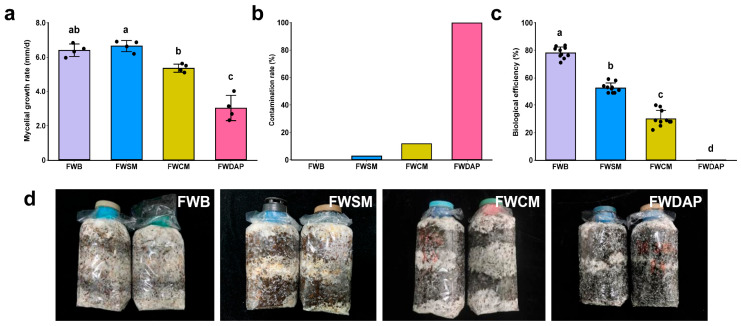
Effects of different treatments on the cultivation of *Pleurotus ostreatus*. (**a**) Mycelial growth rate; (**b**) contamination rate; (**c**) biological efficiency; (**d**) morphological features of mycelial growth on day 7 under different treatments. Different lowercase letters indicate significant differences (*p* < 0.05).

In this study, *P. ostreatus* was used as the test cultivar to evaluate the effects of different nitrogen sources on bioconversion efficiency. The FWSM treatment exhibited the fastest mycelial growth rate (Figure 7a). The FWB treatment showed no significant difference compared with the FWSM treatment, whereas the FWDAP treatment exhibited the slowest mycelial growth rate and differed significantly from all organic nitrogen source treatments (*p* < 0.05) (Figure 7a,d). After mycelial colonization of the substrate in the FWDAP treatment, mold contamination gradually occurred, ultimately reaching a contamination rate of 100% (Figure 7b). This phenomenon may be related to a limited temperature increase during composting, whereby the pile temperature failed to reach the thermophilic phase, making it difficult to effectively eliminate harmful microorganisms. Reynolds et al. [95] confirmed that insufficient maintenance of the thermophilic phase during composting can lead to the accumulation of pathogenic microbial residues, thereby compromising compost safety. Wang et al. [96] reported that delayed temperature increases and an insufficient duration of the thermophilic phase during composting significantly inhibit organic matter degradation efficiency. In the FWDAP treatment, complex organic components (cellulose, hemicellulose, and lignin) were not effectively degraded, resulting in a scarcity of readily available nutrients for mycelial utilization. The resulting slow mycelial growth rendered the substrate vulnerable to competition from other microorganisms, such as molds, ultimately hindering subsequent fruiting body development. Biological efficiency analysis (Figure 7c) showed that the FWB treatment achieved the highest biological efficiency, followed by the FWSM and FWCM treatments. In contrast, the FWDAP treatment exhibited a biological efficiency of zero due to 100% contamination. Notably, the FWSM treatment displayed relatively low biological efficiency, indicating that rapid mycelial growth does not necessarily translate into increased biological efficiency.

**Figure 8:**
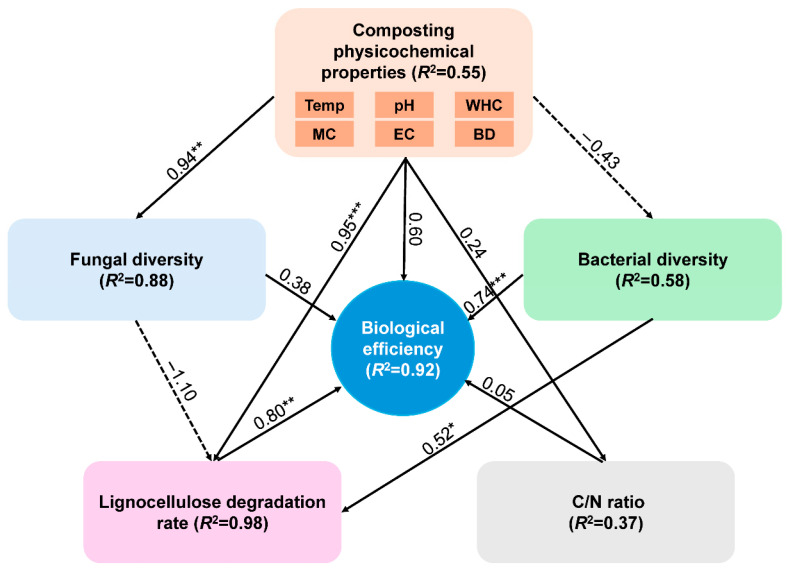
Structural equation modeling (SEM) disentangling the major pathways through which different treatments influence the biological efficiency of *P. ostreatus*. Significant effects are denoted by “*”, “**” and “***”, which represent *p* < 0.05, *p* < 0.01 and *p* < 0.001.

The objective of this study was to elucidate the mechanisms by which different nitrogen sources regulate microbial community structure during composting and to further clarify their regulatory effects on the conversion of forestry waste into a cultivation substrate for oyster mushrooms. A structural equation model (SEM) was employed to quantitatively analyze the potential impact pathways of compost physicochemical properties, bacterial diversity, fungal diversity, lignocellulose degradation rate, and the C/N ratio on the biological efficiency of *P. ostreatus* (Figure 8). The results showed that compost physicochemical properties exerted a highly significant positive effect on fungal diversity and the lignocellulose degradation rate, consistent with previous studies [97,98]. These findings indicate that compost physicochemical properties play a central role in regulating microbial community succession and substrate degradation and indirectly affect the biological efficiency of *P. ostreatus* cultivation [37]. The model further verified that the lignocellulose degradation rate and bacterial diversity exerted highly significant effects on biological efficiency, with bacterial diversity also showing a significant regulatory effect on the lignocellulose degradation rate. Liu et al. [37] reported that compost prepared using corn cobs with an initial particle size of 5 mm exhibited higher bacterial diversity, accompanied by enhanced lignocellulose degradation efficiency and improved *P. ostreatus* biological efficiency. These results suggest that bacterial diversity may indirectly improve *P. ostreatus* biological efficiency by promoting lignocellulose degradation. Effective lignocellulose degradation converts complex organic matter in forestry waste into small-molecule nutrients that are readily available for utilization by *P. ostreatus* mycelia. Ultimately, differences in substrate nutrient supply efficiency exert a significant influence on mycelial growth and the biological efficiency of *P. ostreatus* [24].

## 4. Conclusions

The results of this study demonstrated that treatments with organic nitrogen sources (FWB, FWSM, and FWCM) exhibited a more rapid increase in composting temperature, higher lignocellulose degradation rates, and a greater nutrient supply capacity of the substrate after composting. In contrast, the FWDAP treatment produced lower-quality substrates, primarily due to the absence of an effective thermophilic phase and insufficient degradation of complex organic matter during the composting process. The type of nitrogen source significantly influenced the community composition and diversity of both bacterial and fungal populations. Moreover, treatments with organic nitrogen sources were more likely to develop structurally stable, clearly modular microbial networks with higher abundances of core functional communities. By contrast, the microbial community in the inorganic nitrogen source treatment (FWDAP) exhibited high volatility, characterized by a lower abundance of lignocellulose-degrading groups and reduced functional redundancy related to lignocellulose degradation. During *P. ostreatus* cultivation, organic nitrogen source treatments supported rapid mycelial growth, with the bran-based treatment achieving the highest biological efficiency. This study demonstrated that different nitrogen sources significantly influence compost quality and *P. ostreatus* cultivation performance by regulating microbial community diversity, symbiotic network stability, and lignocellulose degradation efficiency during composting. These findings provide novel insights into the selection of high-quality nitrogen sources for mushroom substrate composts and the optimization of forestry waste bioconversion technologies. However, the study also has certain limitations: the nitrogen source addition ratio was set at a single level, and microbial functions were not directly validated. Future research could further optimize large-scale composting processes, explore the optimal ratio of composite nitrogen sources, and employ multi-omics technologies to gain a deeper understanding of microbial functional mechanisms.

## Data Availability

The original contributions presented in this study are included in the article/Supplementary Material. Further inquiries can be directed to the corresponding authors.

## Supplementary Materials

The following supporting information can be downloaded at: https://www.mdpi.com/article/10.3390/foods15061084/s1, Table S1. Contents of organic matter, total nitrogen, and the C/N ratio in raw materials; Table S2. C/N ratio of the substrate on composting day 1 (D1), composting day 6 (D6), and at the mycelium fully colonized stage (M); Table S3. Alpha diversity indices of bacterial communities during the composting process. Different lowercase letters within the same column indicate significant differences (*p* < 0.05); Table S4. Alpha diversity indices of fungal communities during the composting process. Different lowercase letters indicate significant differences (*p* < 0.05); Table S5. Effects of different treatments on the topological indices of bacterial co-occurrence networks; Table S6. Effects of different treatments on the topological indices of fungal co-occurrence networks; Figure S1. Variations in the composition of predicted bacterial functions during the composting process, inferred using PICRUSt2. (a) Pathway level 1 functional categories; (b) carbohydrate metabolism at pathway level 3; Figure S2. Variations in the composition of fungal functional groups during the composting process, inferred using FUNGuild.
