# Supplementary material for: Microbial Community Dynamics Driven by Different Nitrogen Sources During Forestry Waste Composting for Pleurotus ostreatus Cultivation
Source: Foods. 2026 Mar 20;15(6):1084. doi: 10.3390/foods15061084 (PMC13025609; doi:10.3390/foods15061084)
Supplement: Supplementary file 1 [file foods-15-01084-s001.zip › foods-4180507-supplementary.pdf]

Supplementary Materials:

Table S1. Contents of organic matter, total nitrogen, and the C/N ratio in raw materials.

Material types	Organic matter (%)	Total nitrogen (%)	C/N ratio
Forestry waste	92.2	0.81	101.99
Bran	95.1	2.97	18.57
Soybean meal	94.6	7.72	7.10
Chicken manure	59.6	2.10	6.58
Diammonium phosphate (DAP)	-	18.00	-

Table S2. C/N ratio of the substrate on composting day 1 (D1), composting day 6 (D6), and at the mycelium fully colonized stage (M).

Treatment	D1	D6	M
FWB	69.53	81.91	56.89
FWSM	49.34	57.13	39.18
FWCM	93.39	72.51	50.08
FWDAP	78.31	63.30	56.35

Table S3. Alpha diversity indices of bacterial communities during the composting process. Different lowercase letters within the same column indicate significant differences ($p < 0.05$).

Index	Treatment	D1	D2	D3	D4	D5	D6
Ace	FWB	372.73 ± 77.14 b	455.35 ± 62.65 b	644.72 ± 5.67 ab	599.84 ± 97.60 ab	616.78 ± 103.53 a	576.26 ± 6.39 a
	FWSM	285.47 ± 68.58 b	272.37 ± 30.74 b	332.30 ± 54.14 c	468.15 ± 3.11 b	377.39 ± 15.17 b	424.88 ± 59.63 b
	FWCM	332.27 ± 69.04 b	392.49 ± 86.76 b	466.81 ± 81.99 bc	557.93 ± 83.42 ab	605.97 ± 121.51 a	548.27 ± 43.85 a
	FWDAP	604.82 ± 34.96 a	739.32 ± 96.64 a	745.53 ± 184.03 a	686.26 ± 30.83 a	656.06 ± 57.33 a	586.48 ± 28.01 a
Chao1	FWB	332.92 ± 30.13 b	475.49 ± 60.04 b	545.82 ± 36.09 ab	584.45 ± 41.93 b	581.32 ± 70.68 a	557.05 ± 10.10 a
	FWSM	236.85 ± 29.02 c	257.54 ± 16.40 c	338.70 ± 56.91 b	428.27 ± 23.17 c	379.99 ± 7.12 b	416.76 ± 32.93 b
	FWCM	249.61 ± 22.11 c	378.23 ± 61.82 bc	452.74 ± 40.90 b	535.10 ± 32.00 bc	592.05 ± 94.64 a	559.26 ± 54.11 a
	FWDAP	612.84 ± 39.32 a	728.58 ± 49.82 a	729.39 ± 158.84 a	706.31 ± 63.37 a	664.64 ± 70.40 a	597.59 ± 20.64 a
Shannon	FWB	3.43 ± 0.13 b	4.10 ± 0.11 a	3.92 ± 0.17 ab	4.30 ± 0.06 ab	4.48 ± 0.11 a	4.52 ± 0.04 a
	FWSM	3.16 ± 0.10 b	3.26 ± 0.06 b	3.60 ± 0.27 b	3.84 ± 0.12 b	3.89 ± 0.07 b	3.82 ± 0.05 b
	FWCM	3.33 ± 0.22 b	3.80 ± 0.22 ab	3.89 ± 0.32 ab	3.82 ± 0.51 b	4.41 ± 0.09 a	4.13 ± 0.29 ab
	FWDAP	4.59 ± 0.28 a	4.38 ± 0.37 a	4.49 ± 0.30 a	4.72 ± 0.03 a	4.59 ± 0.16 a	4.06 ± 0.15 b
Simpson	FWB	0.08 ± 0.02 a	0.04 ± 0.00 b	0.07 ± 0.01 a	0.03 ± 0.00 a	0.02 ± 0.00 b	0.02 ± 0.00 a
	FWSM	0.08 ± 0.01 a	0.10 ± 0.01 a	0.07 ± 0.03 a	0.05 ± 0.00 a	0.05 ± 0.00 a	0.06 ± 0.00 a
	FWCM	0.07 ± 0.02 a	0.05 ± 0.01 b	0.07 ± 0.04 a	0.09 ± 0.07 a	0.03 ± 0.00 b	0.06 ± 0.03 a
	FWDAP	0.02 ± 0.01 b	0.04 ± 0.02 b	0.04 ± 0.02 a	0.02 ± 0.00 a	0.03 ± 0.01 b	0.05 ± 0.02 a

Table S4. Alpha diversity indices of fungal communities during the composting process. Different lowercase letters indicate significant differences ($p < 0.05$).

Index	Treatment	D1	D2	D3	D4	D5	D6
Ace	FWB	252.69±18.29a	266.59±63.03ab	275.10±30.50a	272.58±16.77a	273.64±27.67a	320.83±26.44a
	FWSM	295.33±93.66a	309.86±30.70a	321.85±40.76a	307.93±19.44a	315.20±21.47a	289.50±18.10a
	FWCM	311.30±21.33a	322.40±48.17a	326.59±26.52a	308.99±54.48a	299.76±17.00a	286.94±17.42a
	FWDAP	297.63±17.11a	184.16±21.45b	136.20±5.48b	154.14±42.89b	186.90±33.29b	134.22±52.06b
Chao1	FWB	267.12 ± 26.13 a	255.36 ± 38.24 ab	274.51 ± 34.20 a	278.60 ± 16.89 a	263.14 ± 29.72 a	318.44 ± 18.42 a
	FWSM	284.66 ± 75.55 a	302.18 ± 37.04 a	313.09 ± 35.20 a	324.18 ± 39.08 a	308.96 ± 21.04 a	289.52 ± 26.14 a
	FWCM	315.53 ± 19.87 a	318.09 ± 53.13 a	326.02 ± 19.06 a	303.83 ± 54.50 a	303.40 ± 19.01 a	287.91 ± 27.72 a
	FWDAP	272.67 ± 25.81 a	146.00 ± 41.44 b	117.39 ± 13.37 b	133.60 ± 31.34 b	131.70 ± 26.30 b	118.25 ± 39.77 b
Shannon	FWB	2.41 ± 0.27 b	2.62 ± 0.10 a	2.84 ± 0.23 a	2.80 ± 0.25 a	2.50 ± 0.72 ab	2.95 ± 0.25 a
	FWSM	2.45 ± 0.16 b	2.54 ± 0.48 a	2.89 ± 0.26 a	2.87 ± 0.06 a	2.86 ± 0.08 a	3.06 ± 0.08 a
	FWCM	3.33 ± 0.13 a	2.98 ± 0.20 a	3.13 ± 0.18 a	2.93 ± 0.33 a	3.38 ± 0.10 a	2.91 ± 0.55 a
	FWDAP	2.22 ± 0.37 b	0.94 ± 0.20 b	1.88 ± 0.19 b	2.01 ± 0.35 b	1.58 ± 0.54 b	1.70 ± 0.45 b
Simpson	FWB	0.17 ± 0.04 a	0.14 ± 0.02 b	0.10 ± 0.03 ab	0.11 ± 0.03 a	0.19 ± 0.18 a	0.10 ± 0.05 ab
	FWSM	0.15 ± 0.02 ab	0.14 ± 0.08 b	0.10 ± 0.02 b	0.10 ± 0.01 a	0.10 ± 0.01 a	0.09 ± 0.01 b
	FWCM	0.07 ± 0.01 b	0.11 ± 0.03 b	0.10 ± 0.04 ab	0.13 ± 0.05 a	0.07 ± 0.01 a	0.14 ± 0.08 ab
	FWDAP	0.20 ± 0.05 a	0.63 ± 0.07 a	0.24 ± 0.09 a	0.23 ± 0.10 a	0.41 ± 0.21 a	0.33 ± 0.15 a

Table S5. Effects of different treatments on the topological indices of bacterial co-occurrence networks.

Network indices	FWB	FWSM	FWCM	FWDAP
Number of nodes	373	238	348	463
Number of edges	4027	2148	3732	6167
Positive edges	3849	1973	3623	5972
Negative edges	178	175	109	195
Average degree	21.59	18.05	21.45	26.64
Average path length	4.08	4.58	4.32	5.17
Network diameter	11.18	14.98	15.90	14.07
Network density	0.06	0.08	0.06	0.06
Clustering coefficient	0.66	0.77	0.71	0.86
Average clustering coefficient	0.68	0.78	0.73	0.70
Average weighted degree	20.68	17.45	20.59	25.75
Modularity	0.58	0.67	0.61	0.59

Table S6. Effects of different treatments on the topological indices of fungal co-occurrence networks.

Network indices	FWB	FWSM	FWCM	FWDAP
Number of nodes	180	227	216	148
Number of edges	1258	838	1671	6195
Positive edges	1238	838	1661	6194
Negative edges	20	0	10	1
Average degree	13.80	7.38	15.47	83.72
Average path length	1.79	3.31	3.19	1.08
Network diameter	6.55	9.13	12.24	2.82
Network density	0.08	0.03	0.07	0.57
Clustering coefficient	0.90	0.74	0.83	0.98
Average clustering coefficient	0.80	0.78	0.76	0.94
Average weighted degree	13.57	6.64	14.99	82.57
Modularity	0.74	0.78	0.58	0.02

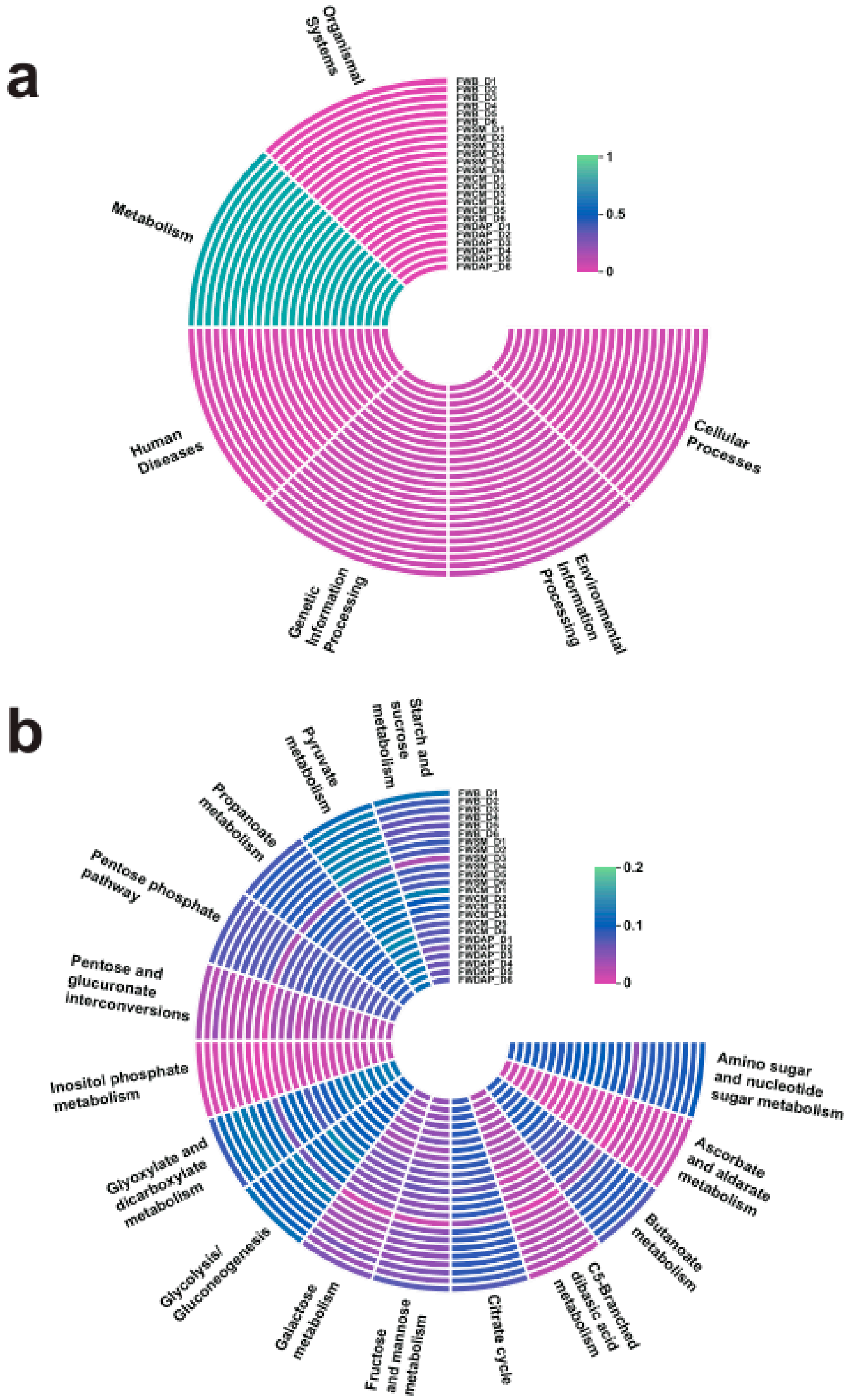

Figure S1. Variations in the composition of predicted bacterial functions during the composting process, inferred using PICRUSt2. (a) Pathway level 1 functional categories; (b) carbohydrate metabolism at pathway level 3.

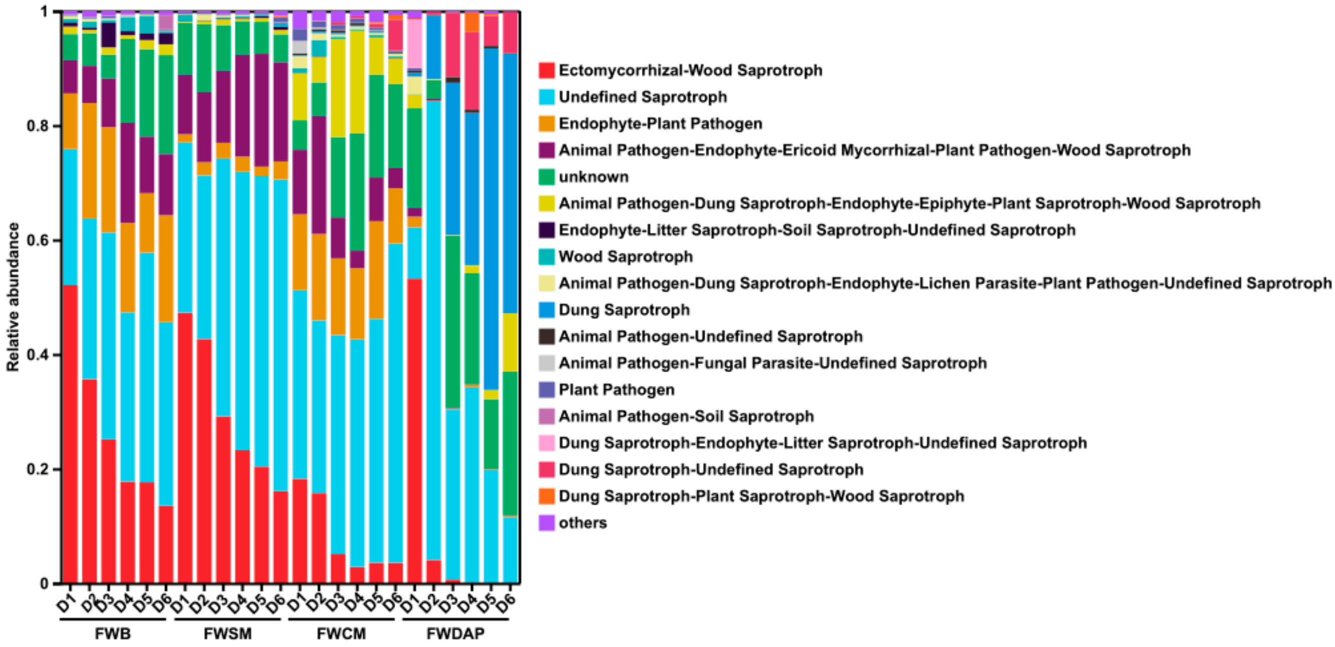

Figure S2. Variations in the composition of fungal functional groups during the composting process, inferred using FUNGuild.
